# Genome-Wide Identification and Expression Analysis of Calmodulin-Like Gene Family in *Paspalums vaginatium* Revealed Their Role in Response to Salt and Cold Stress

**DOI:** 10.3390/cimb45020109

**Published:** 2023-02-16

**Authors:** Meizhen Yang, Jingjin Chen, Tingting Liu, Leilei Xiang, Biao-Feng Zhou

**Affiliations:** 1 Guangdong Engineering Research Center for Grassland Science, State Key Laboratory for Conservation and Utilization of Subtropical Agro-Bioresources, College of Life Sciences, South China Agricultural University, Guangzhou 510642, China; 2 Key Laboratory of Plant Resources Conservation and Sustainable Utilization, South China Botanical Garden, Chinese Academy of Sciences, Guangzhou 510650, China

**Keywords:** *Paspalum vaginatum*, calmodulin-like, abiotic stress, salt stress, cold stress, tandem duplication

## Abstract

The calmodulin-like (CML) family is an important calcium (Ca^2+^) sensor in plants and plays a pivotal role in the response to abiotic and biotic stresses. As one of the most salt-tolerant grass species, *Paspalums vaginatum* is resistant to multiple abiotic stresses, such as salt, cold, and drought. However, investigations of PvCML proteins in *P. vaginatum* have been limited. Based on the recently published *P. vaginatum* genome, we identified forty-nine PvCMLs and performed a comprehensive bioinformatics analysis of PvCMLs. The main results showed that the PvCMLs were unevenly distributed on all chromosomes and that the expansion of PvCMLs was shaped by tandem and segmental duplications. In addition, cis-acting element analysis, expression profiles, and qRT–PCR analysis revealed that PvCMLs were involved in the response to salt and cold stress. Most interestingly, we found evidence of a tandem gene cluster that independently evolved in *P. vaginatum* and may participate in cold resistance. In summary, our work provides important insight into how grass species are resistant to abiotic stresses such as salt and cold and could be the basis of further gene function research on CMLs in *P. vaginatum*.

## 1. Introduction

Plants are subjected to various biotic and abiotic stresses, such as pathogens, low temperature, salt, and drought stresses [1,2]. As an important second messenger in organisms, calcium (Ca^2+^) plays a crucial role in various signal transduction pathways [3,4]. Increasing evidence shows that a wide range of external stimuli, such as gravity, light, cold, heat, drought, hypoxia, salt, wind, touch, mechanical damage, and pathogen attack, can rapidly induce an increase in the intracellular Ca^2+^ concentration [5,6]. Interestingly, the transient changes in the intracellular Ca^2+^ concentration caused by various stresses, so-called calcium signals [2,7,8], are different in amplitude, duration, frequency, and spatial distribution in cells. Our current knowledge has confirmed that calcium signals are sensed by unique Ca^2+^ sensors or Ca^2+^-binding proteins [9,10]. Ca^2+^-binding proteins binding to Ca^2+^ trigger conformational changes, and the modulation of activity subsequently regulates downstream targets, thereby transmitting Ca^2+^ signals [10,11].

There are three types of Ca^2+^ sensor proteins in plants, including calmodulin (CaM) and calmodulin-like protein (CML), Ca^2+^-dependent protein kinases (CDPK/CPKs), and calcineurin-B-like protein (CBL) [12,13,14]. Among them, CaMs and CMLs do not contain any functional domains except for EF-hand motifs [15]. Previous studies have shown that CaM proteins are highly conserved in all eukaryotes, while CML proteins are only present in plants. The identification of CMLs has been accomplished in several plants, such as *Arabidopsis thaliana* [13], rice [16,17], tomato [18], soybean [19,20], and other species [21,22,23,24,25]. These studies demonstrated that the members of the CML family are quite different in sequence, length, and number of EF-hand motifs [13,26,27]. Moreover, gene expression profiles in multiple species show that the expression patterns of CML genes vary greatly in response to external stimuli and hormones [28]. This evidence highly suggests that the divergent functions are likely to have evolved in CMLs [27]. In model species, the functions of CMLs were reported to participate in the plant developmental process and response to various stresses, such as the accumulation of JA [29], resistance to pathogens [30,31], salinity stress [32,33,34], cold stress [33,35,36], and drought stress [33,34]. These works suggest that CMLs are critical to plant survival. However, until now, the mechanisms of how CMLs regulate different pathways to respond to environmental stimuli have not been fully explored, especially in nonmodel species.

*Paspalums vaginatum* Sw. (seashore paspalum) is a perennial grass species of *Paspalum* L. (Poaceae), and as a warm-season turfgrass, has been widely planted in tropical and subtropical areas [37,38,39,40]. *P. vaginatum* is native to high-salinity regions such as saltwater beaches and shows great salt tolerance to saline environments [37,41,42,43]. Other studies have reported that *P. vaginatum* is also advantageous compared with other grasses due to its excellent tolerance to drought, cold, low light, waterlogging, barren land, and wear [37,41]. All these characteristics make *P. vaginatum* a good model for learning how plants respond to abiotic stimuli, like salt and cold stress. Understanding the molecular mechanisms of how *P. vaginatum* is resistant to environmental stresses could not only help to better utilize the germplasm resource of *P. vaginatum* but also contribute to crop breeding in other crop species, such as rice, *Zea mays*, and *Sorghum bicolor* [44].

Here, we investigated the CML gene family in the salt-tolerant species *P. vaginatum*. We identified 49 PvCML genes in the *P. vaginatum* genome and performed comprehensive bioinformatics analyses, including molecular characterization, phylogenetic classification, and analyses of motif and gene structure, chromosome location, and cis-elements. Our results clearly show that the functions of PvCMLs have largely diverged and that the evolutionary history of PvCMLs was shaped by both tandem and segmental duplications. Moreover, we identified fourteen PvCMLs that respond to salt and cold stresses by integrating the analysis of expression profiles, putative cis-element identification, and qRT–PCR. These results will provide valuable insights into further studies of PvCML genes at the physiological and molecular levels.

## 2. Materials and Methods

### 2.1. Identification of CML Genes in the Paspalum vaginatum Genome

To perform CML gene identification in *P. vaginatum*, we downloaded the *P. vaginatum* genome and nonredundant protein sequences from Phytozome 13 (https://phytozome-next.jgi.doe.gov/info/Pvaginatumv3_1/, accessed on 1 April 2022) [44]. Using the HMM search tool embedded in HMMER v3.3.2 and a hidden Markov model (HMM) profile of EF-hand (PF00036) obtained from the Pfam database (http://pfam.xfam.org/, accessed on 2 April 2022) [45], we searched for potential CML proteins in *P. vaginatum* (e-value < 1 × 10^−5^). For all retrieved potential sequences, we checked the presence of the EF-hand domain using SMART (http://smart.embl-heidelberg.de/, accessed on 20 April 2022) [46] and Pfam. Only those proteins containing EF-hand motifs and not containing other functional motifs were retained for downstream analyses. Additionally, we downloaded 50 CML protein sequences of *A. thaliana* from TAIR (https://www.arabidopsis.org/, accessed on 1 April 2022) and 54 nonredundant CML protein sequences identified by Zhu et al., 2015 [15] and Boonburapong and Buaboocha 2007 [17] for downstream phylogenetic and collinearity analyses.

### 2.2. Sequence Alignment and Phylogenetic Analysis

We performed multiple sequence alignments in MUSCLE v3.8.31 [47] with the default settings for CML protein sequences of *P. vaginatum*, rice, and *A. thaliana* and then manually adjusted the incorrect alignments. For the phylogenetic analysis, we performed neighborhood joining (NJ) analysis in MEGA 11 [48] with the parameter settings of pairwise deletion and 1000 bootstrap replicates. Visualization of the NJ tree was conducted using the online tool Evoview (http://www.evolgenius.info/evolview/, accessed on 15 June 2022) [49].

### 2.3. Characterization and Assessments of Gene Structure and Conserved Domains of PvCML Genes

We predicted the physical and chemical characteristics of molecular weight (MW), theoretical isoelectric point (pI), and grand average of hydropathicity (GRAVY) for the proteins in the ExpasyProtParam server (http://web.expasy.org/protparam/, accessed on 6 June 2022). For gene structure analysis, we extracted exon–intron information from the *P. vaginatum* genome annotation file downloaded from Phytozome 13 and visualized it using in-house R scripts. For conserved domain detection, we performed analyses using both MEME tools [50] and Pfam. The parameters of MEME analysis were set to the minimum width of motifs of 10 and the maximum width of motifs of 40. Visualization of the conserved domains was conducted using TBtools [51].

### 2.4. Collinearity and Chromosomal Locations of PvCML Genes

We retrieved the chromosomal locations of CML genes in *P. vaginatum*, rice, and *A. thaliana* from each genome annotation file (gff3 format). Using MCScanX [52], we performed collinearity analyses to identify the collinear relationship between *P. vaginatum* and rice and between *P. vaginatum* and *A. thaliana* and assessed gene duplication events. Synteny relationships and chromosomal locations of CML genes were visualized using Circos v0.69-8 [53] and JCVI [54]. In addition, we calculated the Ka/Ks for each gene pair using the function yn00 embedded in PAML v4.9 [55].

### 2.5. Analysis of Putative Cis-Acting Elements

We extracted upstream 2-kb genomic sequences of PvCMLs to conduct this analysis. Retrieved nucleic acid sequences were used to identify putative cis-acting elements in the PlantCARE database (http://bioinformatics.psb.ugent.be/webtools/plantcare/html/, accessed on 8 July 2022) [56].

### 2.6. Transcription Expression Analysis of PvCML Genes under the Stress and Cold Treatments

To explore the expression changes of PvCMLs under salt stress conditions, we obtained Illumina RNA-seq data from the NCBI Sequence Read Archive (SRA) with accession number PRJNA395934 [38]. After filtering low-quality bases of reads using Trimmomatic v0.38 [57], we generated a de novo transcription assembly using Trinity v2.14.0 [58]. Using TransDecoder v5.5.0 [59], we identified 92,967 ORFs (47.8% of all trinity transcripts) among 220,886 trinity transcript sequences. After clustering the potential coding genes with a sequence identity ≥95%, we retained 77,735 unigenes for downstream analyses. To identify the differential expression between the two treatments (control and salt-treated), we aligned the trimmed reads to 77,735 unigenes and estimated the FPKM counts for each unigene in each sample with RSEM software [60]. The differential expression analysis was then conducted using the R package DESeq2 [61]. The differentially expressed genes were defined with a threshold of adjusted *p* value ≤ 0.05 and |log2(fold change)| > 1.5. Then, we searched PvCMLs in the unigenes using Blastp [62] with a default parameter, and only unigenes with coverage higher than 95% and identity scores higher than 95% to PvCMLs were recognized as the same gene.

Moreover, we downloaded RNA-seq data from the NCBI Sequence Read Archive (SRA) under accession number PRJNA343268 [63] to explore the expression profile of PvCMLs under 6 °C cold stress conditions. Here, we performed reference-based transcriptome analysis because the length of paired-end reads was too short to ensure the accuracy of de novo assembly (paired-end 50 bp). To perform this analysis, we first trimmed low-quality bases of reads using Trimmomatic, aligned clean reads to the *P. vaginatum* genome using HISAT2 [64], and then evaluated the expression level of PvCMLs using StringTie v2.1.6 [65]. The differential expression analysis was conducted using the R package DESeq2. The differentially expressed genes were determined using the same thresholds as mentioned above.

### 2.7. Plant Materials and Treatments

We generated seashore paspalum cultivars (sea spray) from 2.5 cm diameter plugs and grew the plants in pots containing a mixture of sand and peat. After four weeks of growth, the plants were carefully removed from the pot, washed briefly, and then incubated with Hoagland’s nutrient solution for one week. For salt treatment, the plants were immersed in nutrient solutions containing 200 mM NaCl, and three leaves of paspalum were collected at 0 h, 2 h, and 24 h following treatment. For cold treatment, the plants were immersed in nutrient solutions, and three leaves of paspalum were collected at 0 h, 2 h, and 24 h following 4 °C treatment. Both treatments were performed with three biological replicates, and all harvested samples were frozen in liquid nitrogen immediately after sampling and stored at −80 °C for RNA extraction.

### 2.8. Gene Expression Analysis Using Real-Time PCR

TRIZOL reagent (AG21102, Accurate, Guangzhou, China) was used to extract the total RNA of collected samples, and the PrimeScriptTM RT reagent kit (RR047A, TaKaRa, Dalian, China) was used to synthesize first-strand cDNA according to the manufacturer’s protocol. The gene-specific primers were designed using the online software Primer-3Plus (https://primer3plus.com/, accessed on 8 July 2022). Quantitative real-time PCR (qRT–PCR) was conducted using gene-specific primers (Table S5) in a 20 μL reaction with TB Green^®^ Premix Ex TaqTM Supermix (RR420L, TaKaRa, Dalian, China). The qRT–PCR program was carried out as follows: 40 cycles of denaturation at 95 °C for 30 s, annealing at 55 °C for 10 s, and extension at 72 °C for 34 s. The 2^−ΔΔCt^ method was used to analyze the data. The *Pvactin* gene was used as the internal control [66]. All experiments were performed with three technical replicates and three biological replicates.

## 3. Results

### 3.1. Identification and Characterization of CML Proteins in the P. vaginatum Genome

Based on the EF-hand motif sequence (PF00036) and the HMM search method, we searched for potential calmodulin-like genes in *P. vaginatum* and identified 49 nonredundant CML proteins (Table 1 and Table S1). These proteins were further confirmed using Pfam and SMART to ensure that they did not possess identifiable functional domains other than Ca^2+^-binding motifs. We then designated these PvCMLs as PvCML01 to PvCML49 according to their relative location in the reference genome (Table 1). Of these PvCML proteins, the amino acid length ranged from 83 aa (PvCML34) to 313 aa (PvCML24) with an average of 180 aa, the molecular weights ranged from 8.93 kDa (PvCML34) to 32.46 kDa (PvCML24), and the isoelectric points ranged from 3.87 (PvCML46) to 9.73 (PvCML12) (Table 1). The results indicated a great variety of PvCMLs in seashore paspalum.

### 3.2. Phylogenetic Alignments of CMLs among P. vaginatum, Rice and Aribidopsis

**Figure 1:**
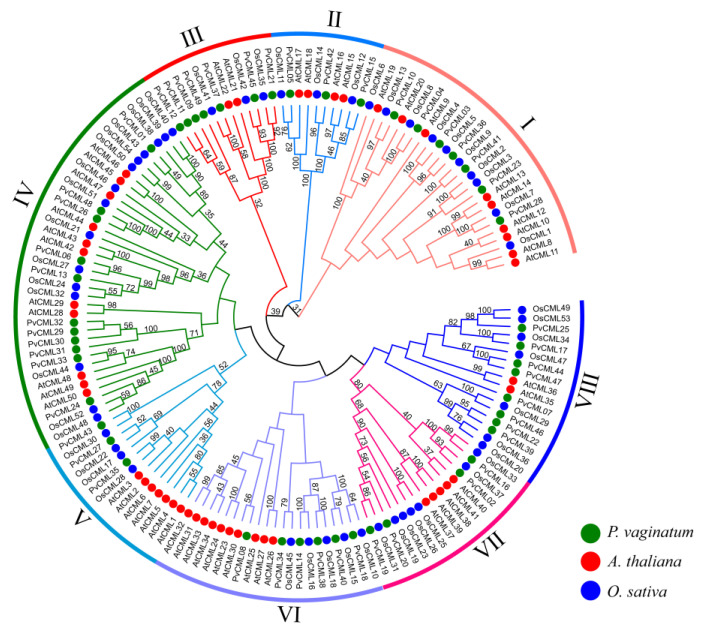
Phylogenetic relationship of calmodulin-like (CML) proteins in *P. vaginatum* (green), *A. thaliana* (red), and rice (blue). An unrooted tree was built using the NJ method in MEGA with 1000 bootstrap replications. The bootstrap values of each node lower than 30 were not shown.

We inferred the phylogenetic relationships of CMLs among *P. vaginatum*, rice, and *A. thaliana* using amino acid sequences of 50 AtCMLs, 54 OsCMLs, and 49 PvCMLs and classified 49 PvCMLs into eight cluster groups (Figure 1). These groups were designated as I to VIII, containing 7, 3, 7, 11, 3, 6, 4, and 8 PvCML genes, respectively. Most PvCMLs had similar proteins in rice (with BS > 70), indicating the potential conserved molecular functions of CMLs between seashore paspalum and rice. However, some gene clusters, such as PvCML09, PvCML11, PvCML12, and PvCML49 in group III and the tandem repeats PvCML29, PvCML30, PvCML31, and PvCML32 in group IV, formed an independent clade, demonstrating that *P. vaginatum* has evolved some novel CML proteins. Considering that seashore paspalum is more tolerant to salt, drought, and cold environments than rice, these novel PvCML proteins may contribute to resistance to abiotic and biotic stresses.

### 3.3. Conserved Motifs and Gene Structure of the PvCMLs

**Figure 2:**
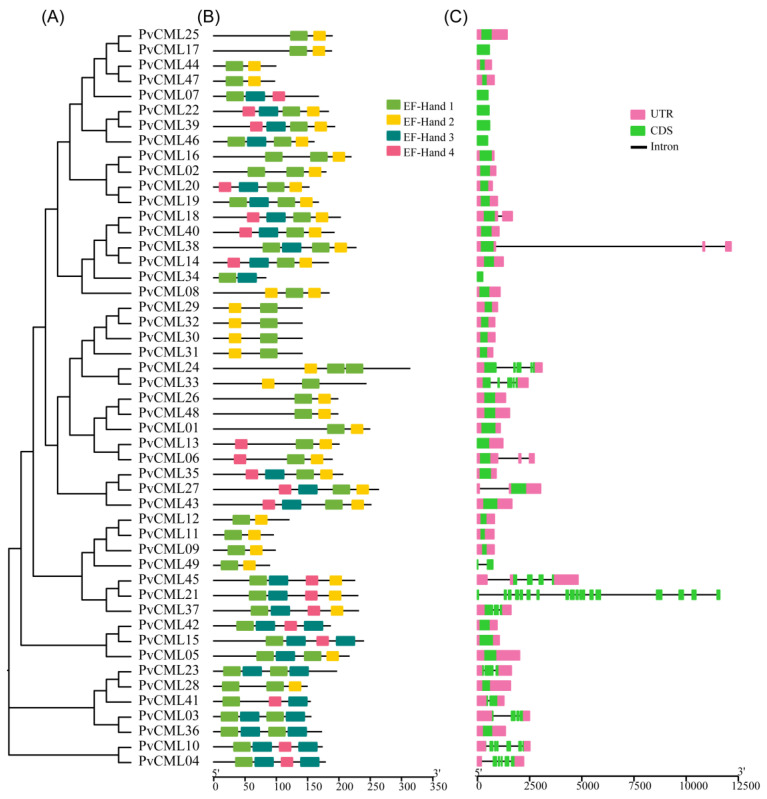
The conserved motif composition and gene structure of CMLs proteins in *P. vaginatum*. (**A**) phylogenetic relationship of PvCMLs. (**B**) The conserved EF-hand motifs of PvCMLs. (**C**) Gene structure of PvCMLs. Pink boxes represent untranslated regions (UTR), green boxes represent coding regions (CDS), and black lines represent introns.

The variety of structural components can provide insight into the corresponding functions and evolutionary relationships of this gene family. The conserved motif analysis revealed that motifs 1 and 2 were present in all PvCML members, demonstrating functional conserved motifs. Of all PvCMLs, nearly half (23 out of 49) contained two pairs of EF-hand motifs, nine contained three EF-hand motifs, and the remaining 16 only contained a pair of EF-hand motifs (Figure 2A). The motif composition of PvCMLs was shown to correspond to their phylogenetic relationships. For example, CML members in group IV mainly contained two conserved motifs, whereas CMLs in groups I, II, V, VI, and VII mainly contained four conserved motifs (Figure 2B). Furthermore, we counted the number of introns among the PvCMLs (Figure 2C). The results showed that the number of introns ranged widely among PvCMLs (from 0 to 17). For most PvCMLs (34/49) without any introns, four PvCMLs contained only one intron, and ten PvCMLs contained 2–6 introns (Figure 2C). PvCMLs with multiple introns mainly belonged to groups I, II, and III, which indicated that the PvCMLs with closer evolutionary relationships have similar gene structures.

### 3.4. Chromosomal Location and Collinearity of PvCMLs

**Figure 3:**
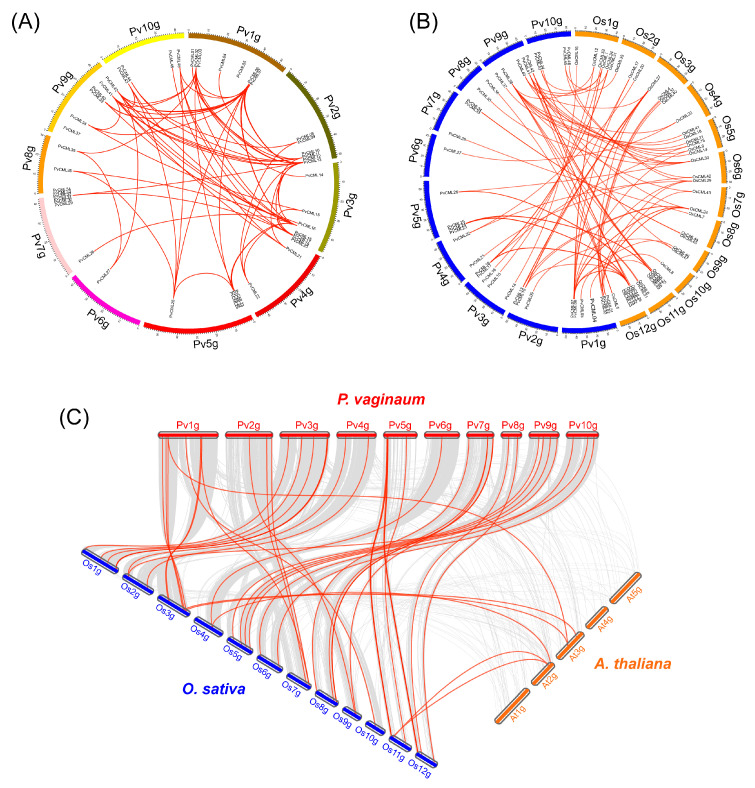
Chromosomal distribution and synteny analysis in the genomes of *P. vaginatum*, *Oryza sativa*, and *A. thaliana*. (**A**) Paralogous genes of PvCMLs in *P. vaginatum*. (**B**) Orthologous CML genes between *P. vaginatum* and rice. (**C**) Orthologous CML genes between *P. vaginatum* and *A. thaliana* and between *P. vaginatum* and rice. Red lines indicate orthologous gene pairs.

**Figure 4:**
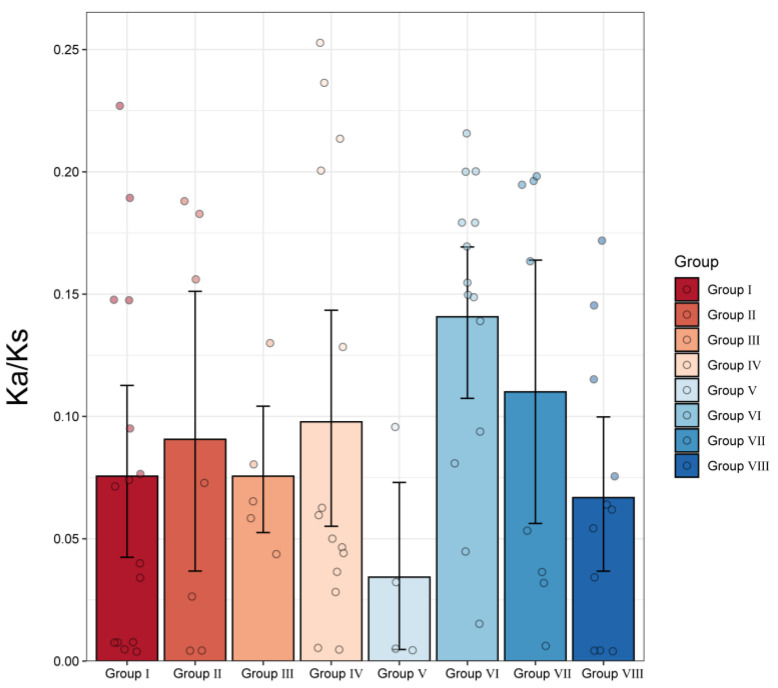
Bar plot of average Ka/Ks ratios for homologous CML gene pairs between rice and *P. vaginatum*. The different colors indicate eight subfamily groups identified in phylogenetic analysis. The error bar is shown in the figure.

We inferred the chromosomal location of PvCMLs based on the genome sequence and genome annotation of *P. vaginatum*. The results clearly showed that the 49 PvCMLs were unevenly distributed on all 10 chromosomes (Figure 3A). For example, chromosomes 1, 7, and 9 contained seven CML genes, whereas chromosome 8 contained only one CML gene (PvCML35). To explore how PvCMLs evolved, we examined the collinearity of PvCMLs within *P. vaginatum*, between *P. vaginatum* and rice and between *P. vaginatum* and *A. thaliana* (Figure 3B,C). The results showed that most PvCML genes included more than one paralog in *P. vaginatum* (Figure 3A, Table S6), indicating that the expansion of PvCMLs is mainly driven by segmental duplications. Twelve CML genes did not have any paralogs, suggesting that these genes may not have experienced duplications or that their duplicated genes were lost during evolution (Table S6). Moreover, we identified two tandem gene arrays (PvCML11-PvCML12 in group III and PvCML29-PvCML32 in group VI) in PvCMLs, demonstrating that the evolutionary processes of PvCMLs were also shaped by tandem duplication. Interestingly, there were no genes in rice and *A. thaliana* that were homologous to these tandem-arrayed genes (Table S7), implying that the tandem genes independently evolved in *P. vaginatum*. Homology prediction and Ka/Ks analysis indicated that PvCMLs were under purifying selection after divergence from rice (Figure 4, Table S7), suggesting that the gene functions of CMLs may be conserved between rice and *P. vaginatum*.

### 3.5. Putative Cis-Element Analysis in Promoter Regions of PvCML Genes

**Figure 5:**
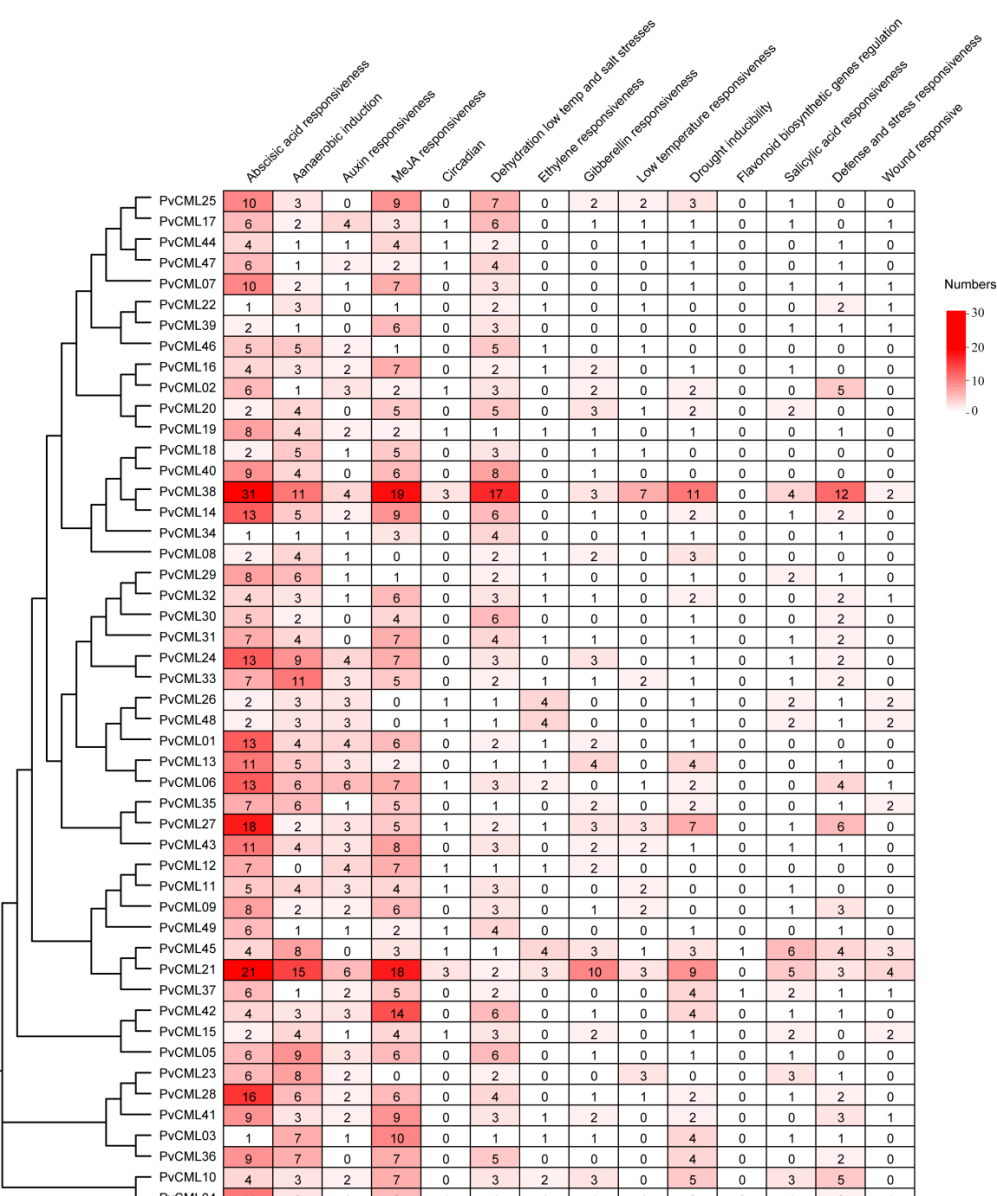
Prediction of cis-acting elements contained in PvCMLs. Analysis of cis-elements was carried out by the PlantCARE database. The analysis showed the presence of 14 cis-elements. The different colors represent the different numbers of cis-elements, with red indicating a high number of cis-elements, while white represents a low number of cis-elements. Values in the boxes represent the number of cis-regulatory elements.

Cis-elements and trans-regulatory factors play critical roles in gene expression. Various hormone response elements and stress response elements have been identified in the promoter regions of the CaM and CML genes in plants [67]. To obtain a better understanding of the functional and transcriptional regulation of PvCML genes, we extracted the upstream 2000 bp sequences of all PvCML genes from the reference genome to identify cis-elements and trans-regulatory factors. The results showed that the hormone response element, stress response element, and other regulatory elements were enriched in the upstream promoter region of PvCMLs (Figure 5, Table S4). The cis-regulatory elements were involved in hormone responses, including auxin, gibberellin, ethylene, salicylic acid, and MeJA. Stress response elements were mainly composed of drought response elements, dehydration low temperature and salt stress response elements, low-temperature response elements, wound responsive elements, and defense and stress response elements. Almost all PvCMLs contained ABA response elements in their promoter regions, with a maximum of 31 in PvCML38 and a minimum of 1 in PvCML3, PvCML22, and PvCML34. In addition, dehydration low temperature and salt stress response elements were widely present in PvCMLs. PvCML38, PvCML21, and PvCML41 were shown to contain 19, 18, and 14 dehydration low-temperature and salt stress response elements, respectively. Most PvCMLs contained MeJA-responsive elements, with PvCML38 and PvCML21 containing 19 and 18, respectively. The flavonoid synthesis gene regulatory elements were found only in PvCML45 and PvCML37. These results indicated that PvCMLs were involved in various abiotic and biotic stresses.

### 3.6. Transcriptome of PvCMLs under Salt and Cold Treatment Conditions

**Figure 6:**
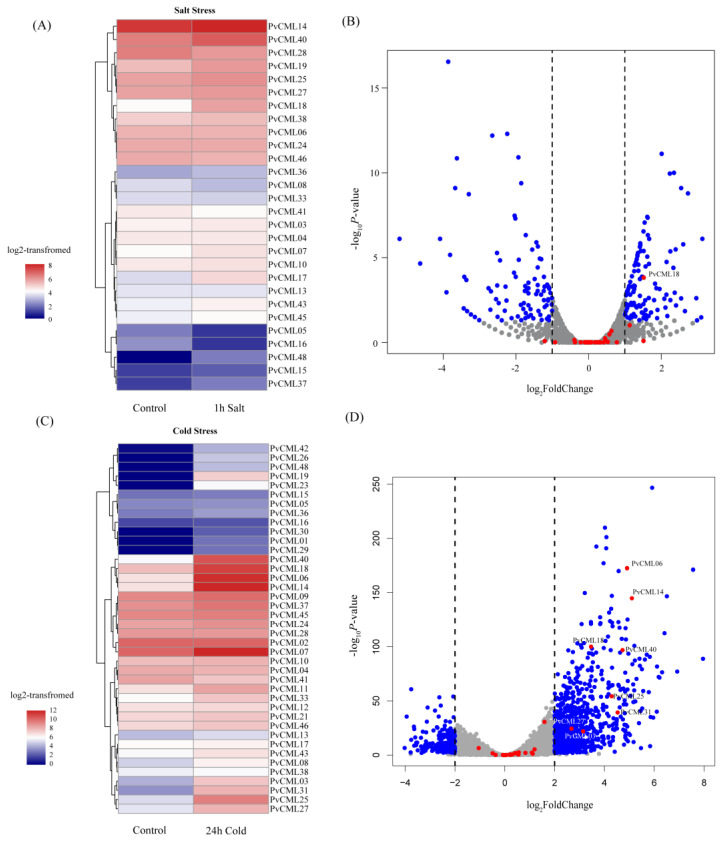
Expression pattern of PvCML genes under salt and cold stress conditions. (**A**). Heatmap of log2–transformed RPKM of PvCML genes after 400 nM NaCl treatment. The colors changing from blue to red indicate the expression level transition from low to high. (**B**). Volcano plot displaying the differentially expressed genes after salt treatment. The blue dots represent genes differentially expressed (adjusted *p* < 0.05) in the 400 nM NaCl treatment group vs. the normal control group. The red dots represent PvCMLs. (**C**). Heatmap of log2–transformed RPKM of PvCML genes after 6 °C cold treatment. The colors changing from blue to red indicate the expression level transition from low to high. (**D**). Volcano plot displaying the differentially expressed genes after 6 °C cold treatment. The blue dots represent genes differentially expressed (adjusted *p* < 0.05) in the 6 °C treatment group vs. the normal control group. The red dots represent PvCMLs.

To gain insights into the putative molecular functions under abiotic stresses, we analyzed the expression pattern of PvCMLs using publicly accessible RNA-seq data. The RNA-Seq data were generated from two different experimental treatments, 400 mM NaCl treatment and 4 °C cold treatment, which reflected the response of PvCMLs to different abiotic stresses. Our results showed that the expression of one PvCML gene was significantly increased under salt treatment (Figure 6A,B, Table S2), and eight PvCMLs were significantly increased under cold treatment (Figure 6C,D, Table S3). Among all differentially expressed genes, PvCML18 increased the gene expression in both salt and cold treatments; PvCML06, PvCML14, PvCML40, PvCML25, and PvCML31 demonstrated a more than two-fold expression level increase under cold treatment, while the expression of PvCML18, PvCML27, and PvCML03 was increased only one-fold.

### 3.7. qRT–PCR of PvCMLs under Salt and Cold Treatment Conditions

**Figure 7:**
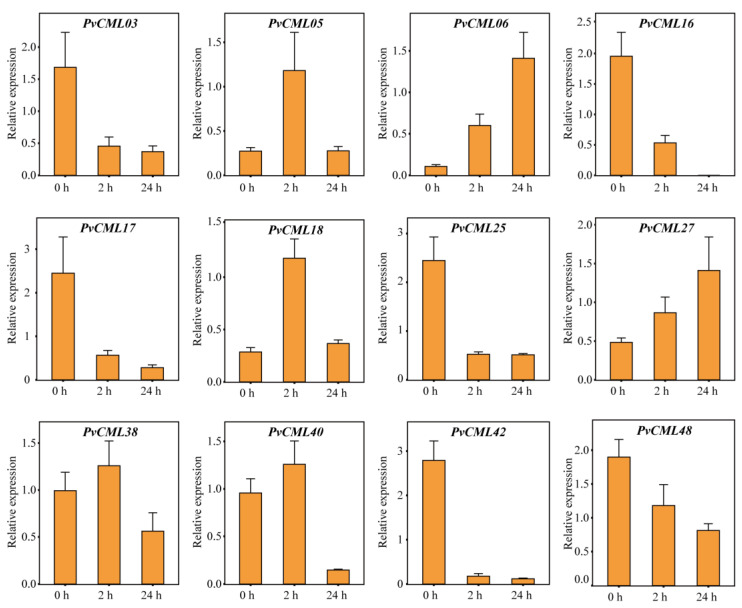
Gene expression analysis by RT–qPCR for the PvCMLs under salt stress conditions. The four-week-old seashore paspalum cultivars (sea spray) were treated with 200 mM NaCl, and three leaves were collected for each sample at 0 h, 2 h, and 24 h following treatment. Three biological replicates were performed for this analysis. The bar plot indicates the relative mRNA expression of each PvCML gene at each treatment time.

**Figure 8:**
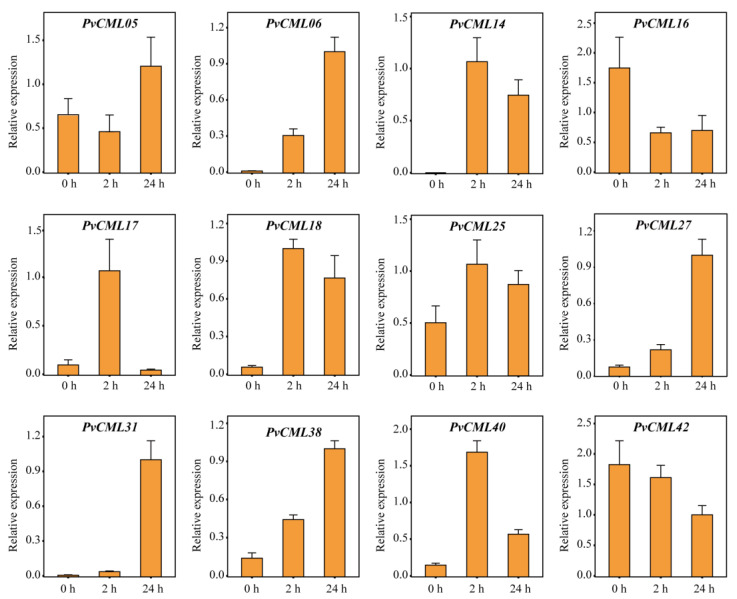
Gene expression analysis by RT–qPCR for the PvCMLs under cold stress conditions. The four-week-old seashore paspalum cultivars (sea spray) were treated with 4 °C, and three leaves were collected for each sample at 0 h, 2 h, and 24 h following treatment. Three biological replicates were performed for this analysis. The bar plot indicates the relative mRNA expression of each PvCML gene at each treatment time.

To further investigate the response of PvCMLs to salt and cold stresses, we estimated the expression levels of PvCMLs using qRT–PCR analysis. Genes with excess “dehydration low temperature and salt stress response” elements (more than six elements) or excess “dehydration low temperature and salt stress response” elements (more than three elements) or genes with differential expression in expression profiles were selected for this analysis. Under salt treatment conditions, the expression of PvCML18 increased at 2 h, identical to the results of the expression profile, while at 24 h, PvCML18 gene expression recovered to the control level (Figure 7). In addition, we found that PvCML06 and PvCML27 expression progressively increased over time, whereas PvCML03, PvCML17, PvCML25, PvCML42, and PvCML48 expression progressively decreased over time (Figure 7 and Figure S2). Under cold treatment conditions, seven differentially expressed genes (PvCML06, PvCML14, PvCML18. PvCML25, PvCML31, PvCML40, and PvCML27) were found to be upregulated in the qRT–PCR analysis, validating the results of the expression profile (Figure 8 and Figure S3). Overall, the qRT–PCR analysis validated the expression profile results and provided new insight into how PvCMLs respond to salt and cold stresses.

## 4. Discussion

Plants have evolved a series of physiological and biochemical mechanisms to cope with environmental and developmental stimuli. One of the most important ways is to recruit Ca^2+^ as a second messenger in response to a given stimulus [68]. During this process, Ca^2+^ sensor proteins play a crucial role in transducing the increase in cytosolic Ca^2+^ concentrations by associating with and altering downstream target proteins [28]. As a member of the Ca^2+^ sensors, the CML family is specific in green plants and crucial in both developmental processes and adaptation to environmental stimuli [15]. In the past decade, the publication of an increasing number of reference genomes has enhanced our ability to identify the members and functions of CMLs in different species. The identification of CML proteins has been accomplished in several plant species, such as rice [15,17], *A. thaliana* [13], maize [22], tomato [18], and other species [20,24,25,69]. However, most of these are model or well-studied species, and the functions and evolutionary processes of the CML family in nonmodel species have not been fully studied. In this study, 49 CML family proteins were identified in one of the most salt-tolerant turfgrass species, *P. vaginatum*. The number of PvCMLs differs from those in rice (54 OsCMLs), maize (46 ZmCMLs), *A. thaliana* (50 AtCMLs), soybean (144 GmCMLs), and grapevine (62 VviCMLs), indicating that the evolutionary processes of PvCMLs are not completely shared with those of other species. Moreover, a comprehensive analysis of PvCMLs, including construction of a phylogenetic tree and analyses of conserved motifs, exon-intron structure, chromosome location, synteny, cis-regulatory elements, interaction network, and expression patterns under salt stress and cold stress conditions was completed. These results provide novel and valuable information that could help us to explore the functions and regulatory mechanisms of CML genes in *P. vaginatum*, especially in salt and cold resistance.

Our results demonstrated that the gene length, theoretical molecular weight (Mw), and isoelectric point (pI) of PvCMLs largely varied (Table 1), which is identical to other species [16,70], indicating that divergent functions are likely to have evolved. Moreover, PvCMLs have less homology with AtCMLs, indicating that CML proteins have highly diverged between monocotyledons and dicotyledons, which is also consistent with the results of other studies [21,71]. The gene location results demonstrated that the PvCMLs were unevenly distributed on all 10 chromosomes, indicating that the evolutionary processes of PvCMLs were shaped by segmental duplication. Several tandem gene arrays, such as PvCMLs29-PvCML32 on chromosome 7 and PvCML11-PvCML12 on chromosome 2, were detected in *P. vaginatum*, suggesting that the evolutionary processes of PvCMLs were also affected by tandem duplications. Additionally, we predicted complex interaction networks among PvCMLs, which indicates the function of PvCMLs associated with each other (Figure S1).

Phylogenetic analysis and collinearity relationships showed that most PvCMLs had homologous genes in rice, suggesting the potential conserved molecular function of CML genes between the two species. All homologous gene pairs were under purifying selection, which further confirmed our inference. In addition, eight PvCMLs were identified to have independently evolved in *P. vaginatum*, and these genes may contribute to adaptation to harsh environments. Four of them (PvCML29-PvCML32) were adjacent to each other, forming a tandem gene array. The expression profile and qRT–PCR analysis demonstrated that the tandem gene array participated in the regulatory process of cold stress (Figure S4), which indicates that the function of this tandem gene array may be associated with cold stimulus. The impact of tandem duplication on plant abiotic and biotic resistance has been reported in multiple taxa [72,73,74,75,76]. Here, we provide another case of the potential role of tandem gene array contributions to resistance to abiotic stress.

The specific expression of genes is determined by the cis-elements in the promoter region. Previous studies have shown that certain CML genes with special cis-acting elements in the promoter region participate in hormonal or abiotic stress responses [67]. For example, low-temperature responsive elements (LTRs) were enriched in the promoter regions of MtCML16 and MtCML33, and the expression of MtCML16 and MtCML33 was significantly induced under cold stress conditions [24]. The promoter activities of AtCML37, AtCML38, and AtCML39 were altered under conditions of drought, salt, and oxidative stresses and mechanical damage, and their transcript expression levels were also significantly increased [77]. In different tissues at different developmental stages of rice, the expression of OsMSR2 was induced by different stresses, such as low temperature, drought, and high temperature [33]. In *P. vaginatum*, PvCMLs were shown to contain various cis-elements related to plant growth and development, abiotic stress, and plant hormones. Almost all PvCML genes were found to contain cis-elements related to abscisic acid responsiveness, indicating that PvCML proteins extensively participate in ABA and drought regulation. Low-temperature and salt-responsive elements were present in most promoters of PvCMLs, suggesting that PvCML proteins are also associated with high salt tolerance. More than half of the PvCMLs contained anaerobic responsive elements (AREs) and MeJA responsive elements, which might be related to the adaptability to the aquatic environment and involvement in the jasmonic acid pathway. The PvCML38 and PvCML21 promoter regions contained almost all cis-acting elements, indicating that PvCML38 and PvCML21 may participate in multiple developmental and stress responses.

Previous studies have demonstrated that CML genes are key regulators involved in salt stress and cold stress. For example, AtCML24 was induced after cold treatment in *A. thaliana* [78]. AtCML9 was associated with salt and low-temperature treatments, and the AtCML9 mutants enhanced resistance to salt stress [34]. Soybean GsCML27 was induced by salt stress, and heterologous expression of GsCML27 enhanced tolerance to salt stress in *A. thaliana* [79]. MtCML40 was induced by salt and low-temperature stress and negatively regulated salt tolerance in *M. truncatula* [32]. MtCML42 and MtCML10 were induced by cold treatment, and overexpression in transgenic plants improved cold tolerance [35,36]. Tomato ShCML44 was induced by a variety of abiotic stresses, and overexpression of ShCML44 could improve tolerance to cold and salt stress [80]. All these works in model species clearly show that CMLs can enhance resistance to salt and cold stresses. In *P. vaginatum*, we analyzed the expression patterns of PvCMLs through expression profiles and qRT–PCR and found evidence of PvCMLs in response to salt and cold stresses. Under salt stress conditions, the expression of PvCML06, PvCML18, PvCML27, PvCML38, and PvCML40 increased over time, indicating that these genes played an important role in regulating the salt tolerance of *P. vaginatum*, while PvCML16, PvCML42, and PvCML48 were downregulated under salt stress conditions, indicating that these genes participated in the negative regulation of salt stress. Furthermore, we found the expression of six CML genes (PvCML06, PvCML14, PvCML18. PvCML25, PvCML31, PvCML40, and PvCML27) were significantly induced under cold stress conditions, indicating that PvCMLs also played important roles in the cold tolerance of *P. vaginatum*. In rice, as a homologous gene of PvCML14 (one of the cold-inducing genes), OsCML16 has been proven to interact with OsPi304 to enhance cold tolerance [81], which provides additional evidence for our results. Overall, our work provides important insights into the role of PvCMLs in salt and cold resistance and could provide a good foundation for further molecular function investigation of PvCMLs in the future.

## 5. Conclusions

As one of the most salt tolerance grass species and famous warm-season turfgrass, the mechanism of excellent abiotic stress resistance of *P. vaginatum* is still largely unknown. In this study, we investigated CML gene family in *P. vaginatum* to evaluate the role of CML genes played in response to salt and cold stress. A total of 49 CML proteins were identified in *P. vaginatum*. Among them, the gene length, molecular weight, theoretical isoelectric point, composition of the conserved domain, and gene structure varied greatly, which demonstrated that the functions of PvCMLs have largely diverged. Synteny analysis indicated that the evolutionary history of PvCMLs was largely affected by tandem and segmental duplications. Moreover, the expression profile and qRT–PCR results suggested that PvCMLs participated in regulating the salt and cold tolerance of *P. vaginatum*. Most notably, we found that evidence of a tandem gene array (PvCML29-PvCML32) indicated that evolution of novel CMLs in *P. vaginatum* may be associated with cold stress. Altogether, our work contributes to insights into a deep understanding of the evolution and expression characteristics of PvCMLs, and the findings of a potential stress resistance gene will guide further molecular function investigation of PvCMLs and germplasm resource utilization of *P. vaginatum* in the future. Moreover, understanding how grass species resist abiotic and biotic stresses could pave the road to crop breeding in other closely related crop species, such as rice, *Z. may*, and *S. bicolor*.

## Figures and Tables

**Table 1:** Information of PvCMLs identified in the *P. vaginatium*.

Gene Name	Locus ID	Length (aa)	MW (kDa)	pI	GRAVY	EF-Hand Domain Number
PvCML01	Pavag01G036200	249	26,549.83	4.93	−0.235	2
PvCML02	Pavag01G067400	179	19,525.77	5.66	−0.444	2
PvCML03	Pavag01G085000	155	17,270.08	3.98	−0.588	4
PvCML04	Pavag01G238700	178	20,176.57	4.62	−0.749	4
PvCML05	Pavag01G274800	216	22,865.16	4.54	−0.289	4
PvCML06	Pavag01G365300	189	20,387.82	4.46	−0.297	3
PvCML07	Pavag01G374900	167	17,550.55	4.16	−0.149	3
PvCML08	Pavag02G205000	184	21,026.19	8.76	−0.649	3
PvCML09	Pavag02G205100	98	11,265.6	5.45	−0.738	1
PvCML10	Pavag02G351900	173	19,858.13	4.76	−0.923	4
PvCML11	Pavag02G352100	95	10,825.18	8.18	−0.674	2
PvCML12	Pavag02G352200	120	13,500.42	9.73	−0.479	2
PvCML13	Pavag02G392400	200	21,075.5	4.47	−0.207	3
PvCML14	Pavag03G074400	183	19,175.09	4.48	−0.538	4
PvCML15	Pavag03G187000	239	24,717.86	4.95	−0.145	4
PvCML16	Pavag03G287500	219	23,379.89	4.96	−0.548	2
PvCML17	Pavag03G401100	188	19,570.96	4.88	−0.233	2
PvCML18	Pavag03G401300	202	20,647.08	4.79	−0.125	4
PvCML19	Pavag03G405900	167	17,384.44	4.42	0.061	4
PvCML20	Pavag03G406300	152	16,591.57	4.2	−0.296	4
PvCML21	Pavag04G074900	230	26,143.37	4.59	−0.559	3
PvCML22	Pavag04G268400	183	19,286.47	4.26	−0.245	4
PvCML23	Pavag05G031700	196	22,180.57	4.48	−0.645	4
PvCML24	Pavag05G038100	313	32,468.23	6.42	−0.372	2
PvCML25	Pavag05G038700	189	19,421.47	4.8	−0.243	2
PvCML26	Pavag05G195800	198	22,248.02	4.57	−0.366	2
PvCML27	Pavag06G144700	263	27,960.3	4.78	−0.429	4
PvCML28	Pavag07G020500	149	16,585.77	4.78	−0.34	3
PvCML29	Pavag07G181000	141	15,274.29	4.53	−0.025	2
PvCML30	Pavag07G181100	141	15,343.27	4.48	−0.073	2
PvCML31	Pavag07G181200	141	15,250.15	4.54	−0.063	2
PvCML32	Pavag07G181300	141	15,341.34	4.53	−0.021	2
PvCML33	Pavag07G226900	243	26,137.43	6.07	−0.193	2
PvCML34	Pavag07G229200	83	8931.05	4.29	−0.15	2
PvCML35	Pavag08G065500	206	22,885.43	4.62	−0.7	4
PvCML36	Pavag08G137400	172	18,391.3	4.28	−0.309	4
PvCML37	Pavag09G042600	231	26,890.24	4.95	−0.685	3
PvCML38	Pavag09G088400	227	23,777.51	5.61	−0.493	4
PvCML39	Pavag09G124700	193	20,236.49	4.3	−0.314	4
PvCML40	Pavag09G126600	192	19,711.92	4.7	−0.184	4
PvCML41	Pavag09G185900	154	16,812.8	4.51	−0.355	2
PvCML42	Pavag09G246700	186	19,477.01	4.73	−0.03	4
PvCML43	Pavag10G059200	251	27,068.03	4.51	−0.524	4
PvCML44	Pavag10G085700	99	11,520.98	9.38	−0.513	2
PvCML45	Pavag10G188500	225	25,934.32	4.75	−0.48	4
PvCML46	Pavag10G241600	160	16,085.05	3.87	0.316	3
PvCML47	PavagK054400	97	11,196.58	6.41	−0.635	2
PvCML48	PavagK123100	198	22,203.98	4.57	−0.368	2
PvCML49	PavagK317500	89	10,140.33	6.41	−0.741	2

Note: Abbreviations: MW, molecular weight; pI, isoelectric point; GRAVY, grand average of hydropathicity.

## Data Availability

The transcriptome data that support the findings of this study are available from National Center for Biotechnology Information (https://www.ncbi.nlm.nih.gov/) (accessed on 17 February 2022) with the access number of SRR5876943, SRR5876944, SRR4280403, SRR4280409, SRR4280414, SRR4280420, SRR4280426 and SRR4280427.

## Supplementary Materials

The following supporting information can be downloaded at: https://www.mdpi.com/article/10.3390/cimb45020109/s1. Figure S1: predicted protein-protein interaction network (PPI) of PvCMLs; Figure S2: qRT-PCR results of 14 PvCMLs under salt stress; Figure S3: qRT-PCR results of 14 PvCMLs under cold stress; Figure S4: RT-qPCR results of PvCML29–32 under cold stress; Table S1: Protein sequence of PvCMLs; Table S2: Expression profile under salt stress; Table S3: Expression profile under cold stress; Table S4: The promoter information of PvCMLs genes; Table S5: Sequences of primers used in RT-qPCR; Table S6: Pairs of paralogous PvCML proteins; Table S7: The Ka/Ks value of pairs of homologous proteins between *P. vaginatium* and rice. All code associated with this project is available at GitHub (https://github.com/zzbbf123/PvCMLs, accessed on 7 December 2022).
