# Supplementary material for: Genome-Wide Identification and Expression Analysis of Calmodulin-Like Gene Family in Paspalums vaginatium Revealed Their Role in Response to Salt and Cold Stress
Source: Curr Issues Mol Biol. 2023 Feb 16;45(2):1693–711. doi: 10.3390/cimb45020109 (PMC9954852; doi:10.3390/cimb45020109)
Supplement: Supplementary file 1 [file cimb-45-00109-s001.zip › cimb-2158222-supplementary/supplementary/SupplementaryFigures.pdf]

Supplementary Information

Genome-Wide Identification and Expression Analysis of
Calmodulin-like Gene Family in *Paspalums vaginatium* Revealed their Role
in Response to Salt and Cold Stress

Meizhen Yang *et al.*

Contents:

Supplementary Figures (Figure S1 – S4)

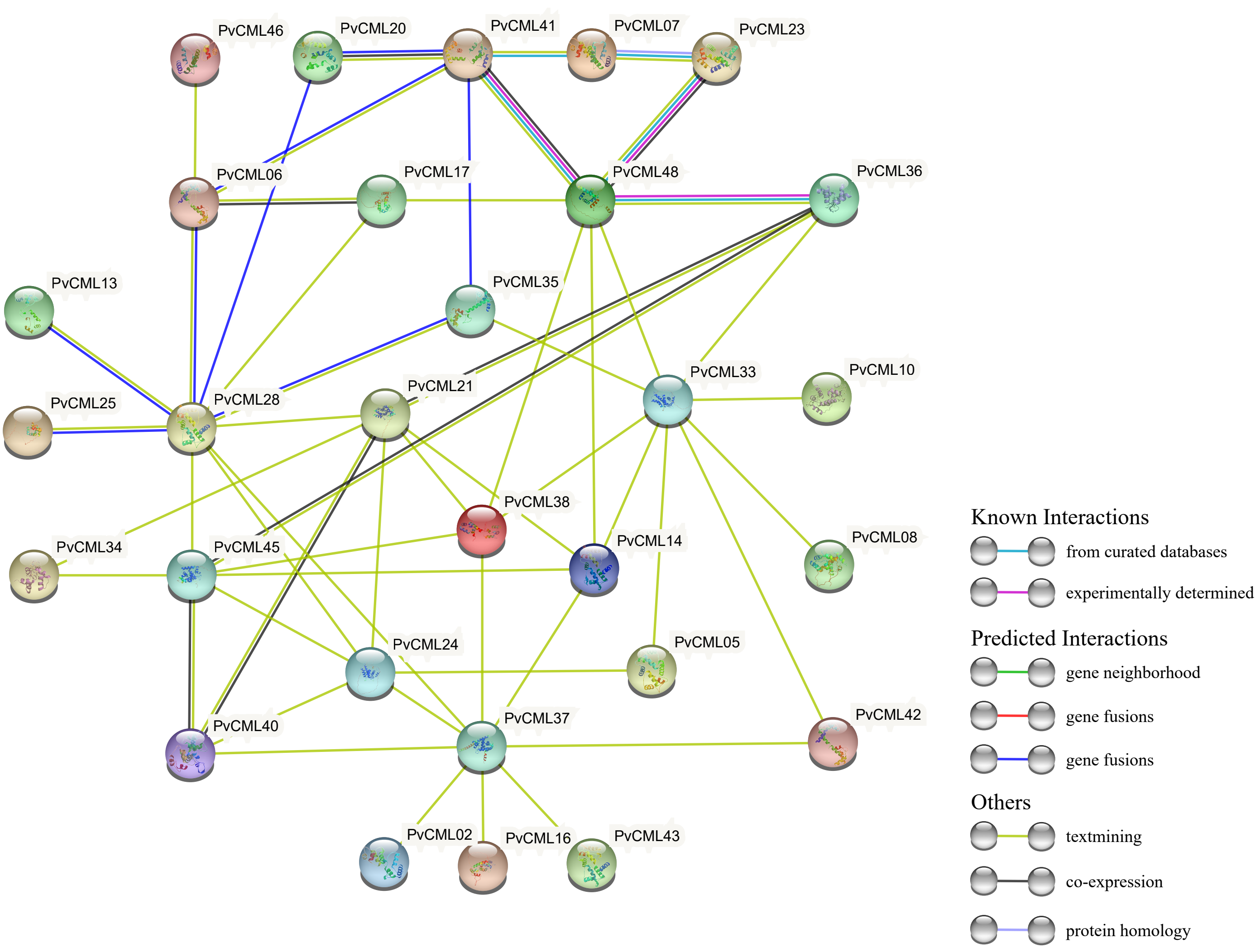

Figure S1. Predicted protein-protein interaction network (PPI) of PvCMLs

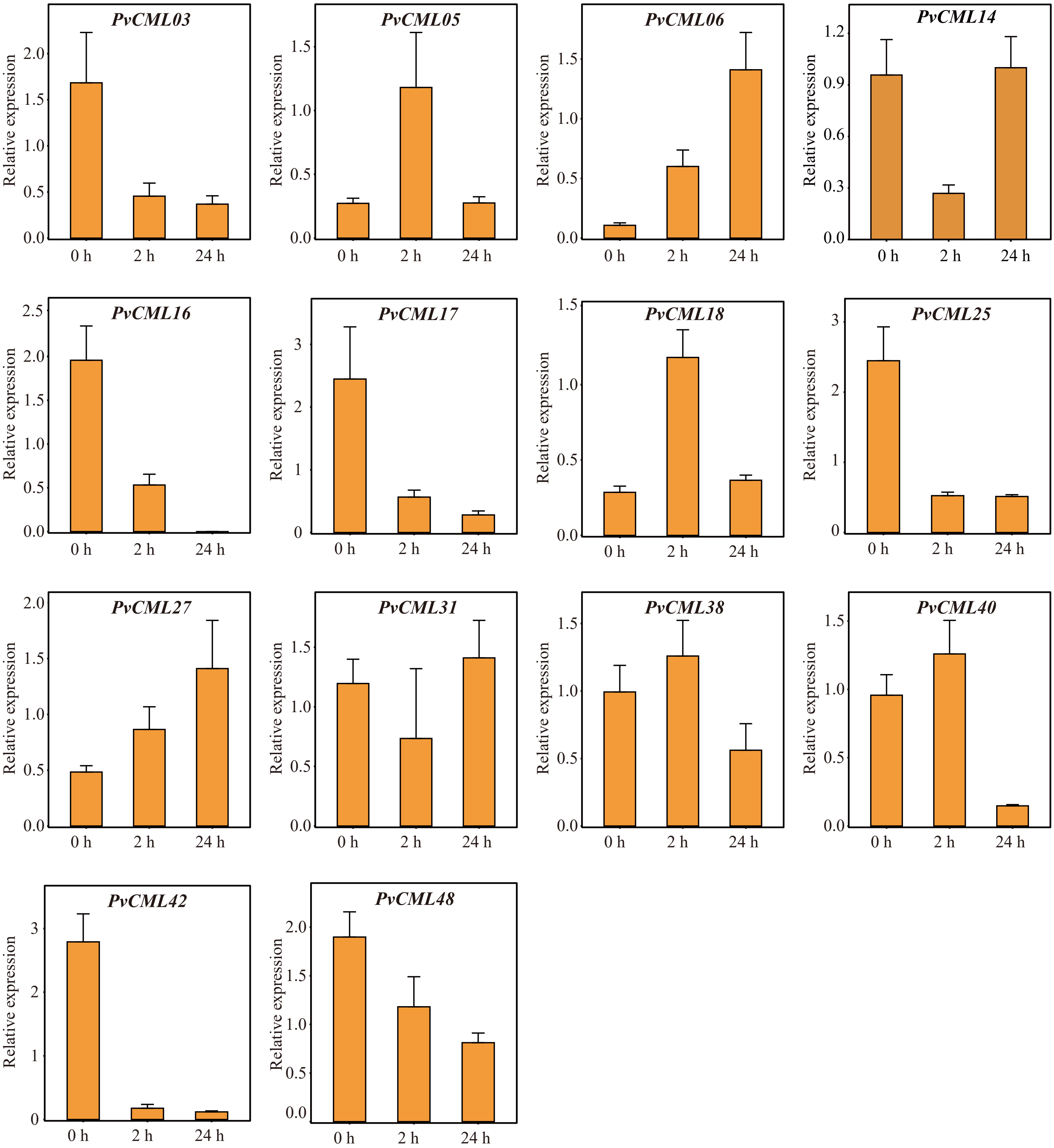

Figure S2. qRT-PCR results of 14 PvCMLs under salt stress

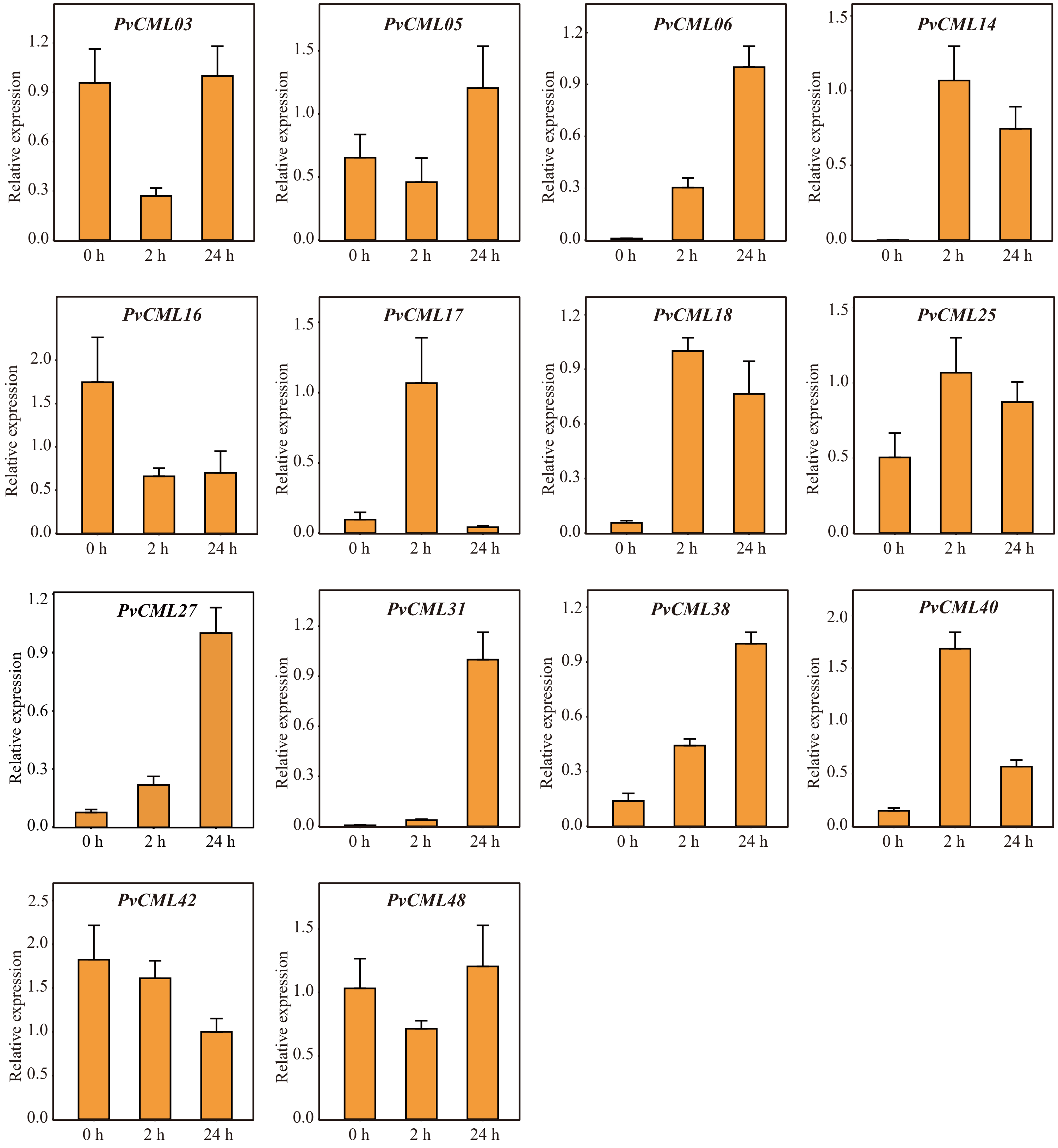

Figure S3. qRT-PCR results of 14 PvCMLs under cold stress

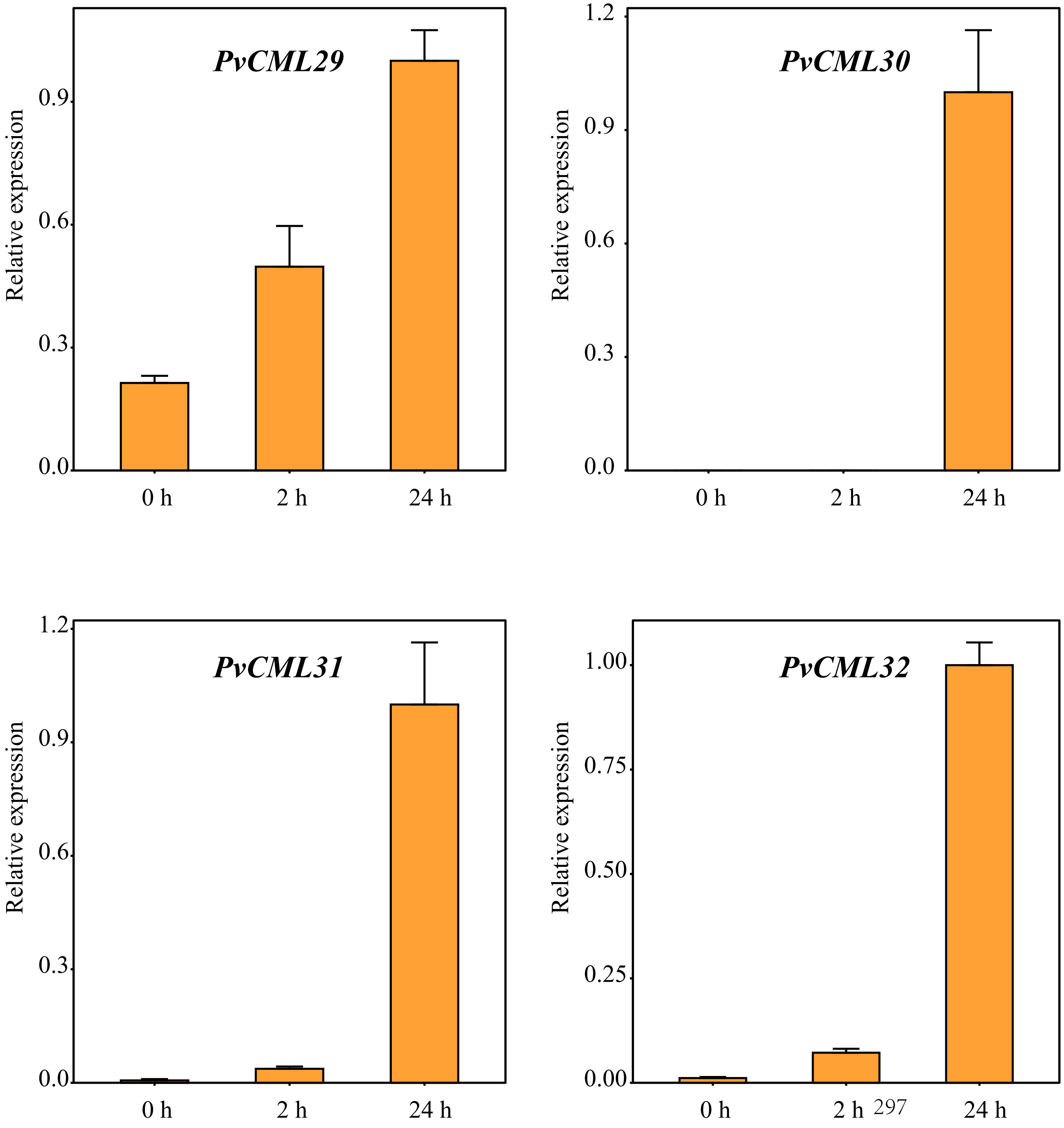

Figure S4. RT-qPCR results of PvCML29-32 under cold stress
